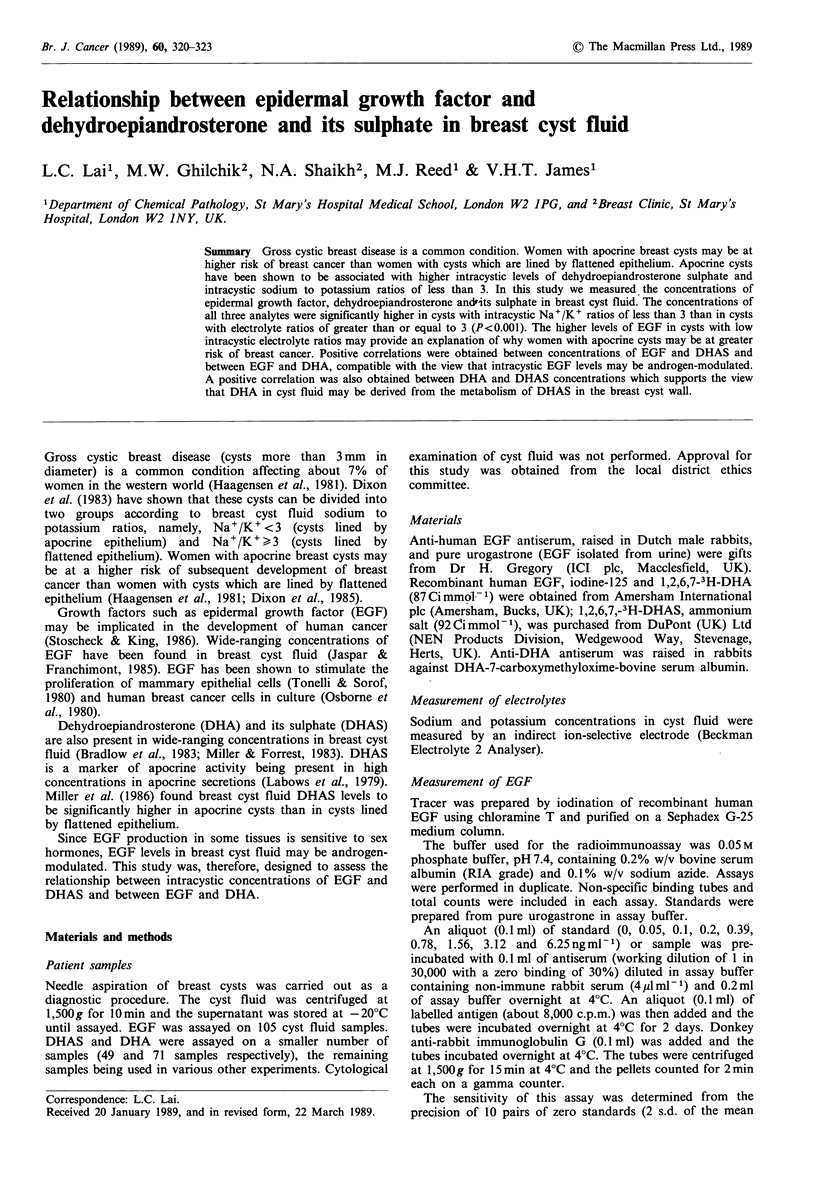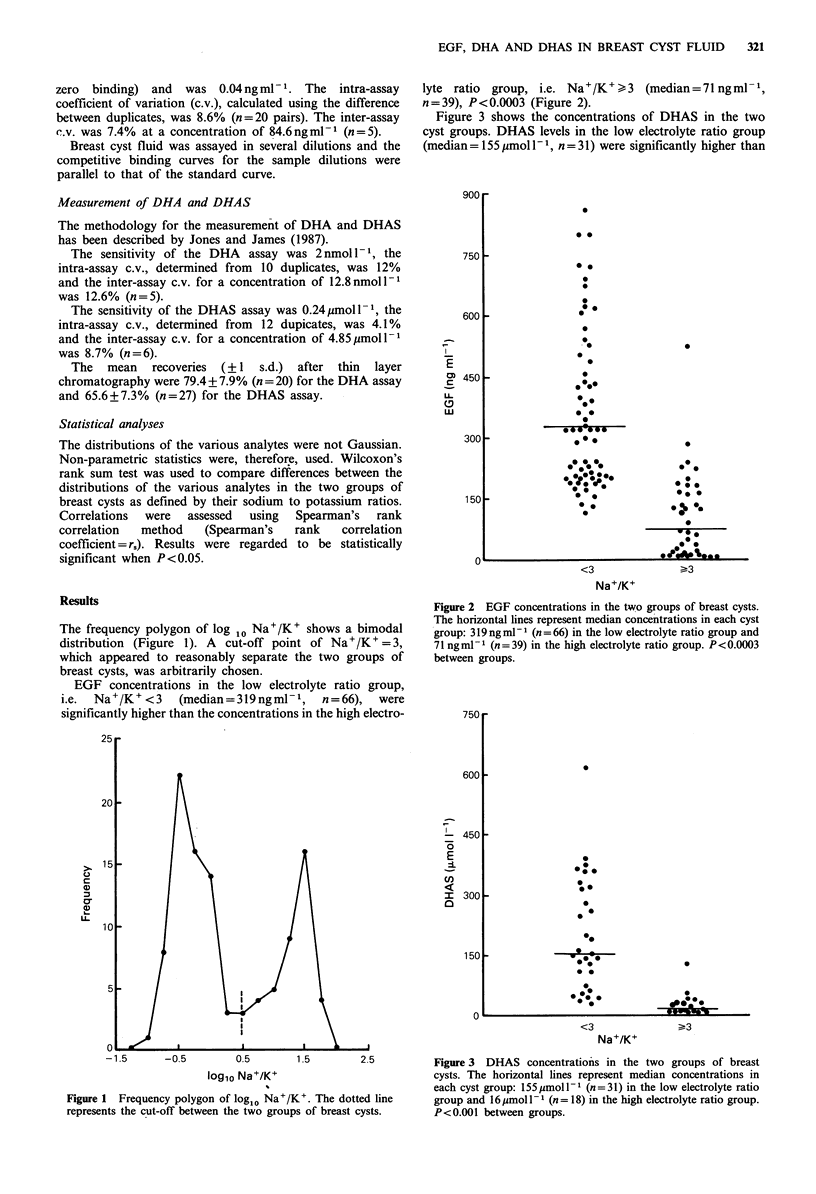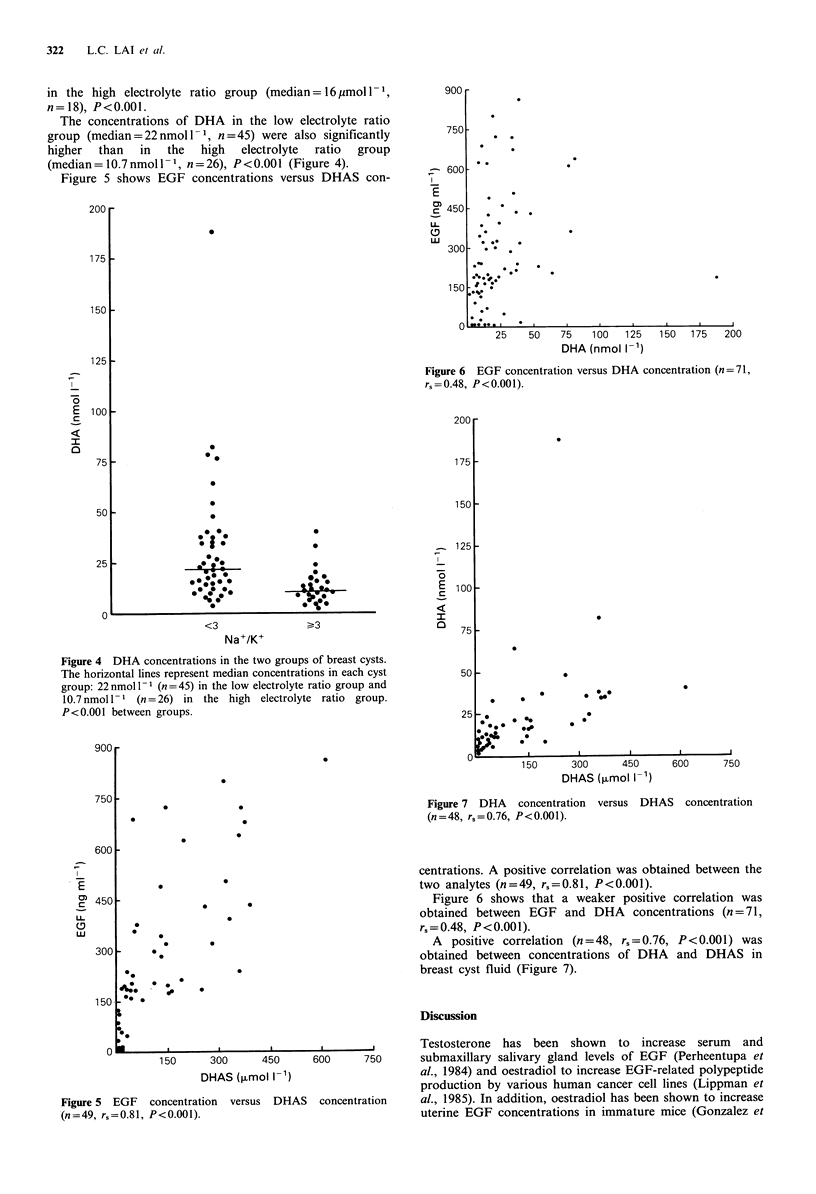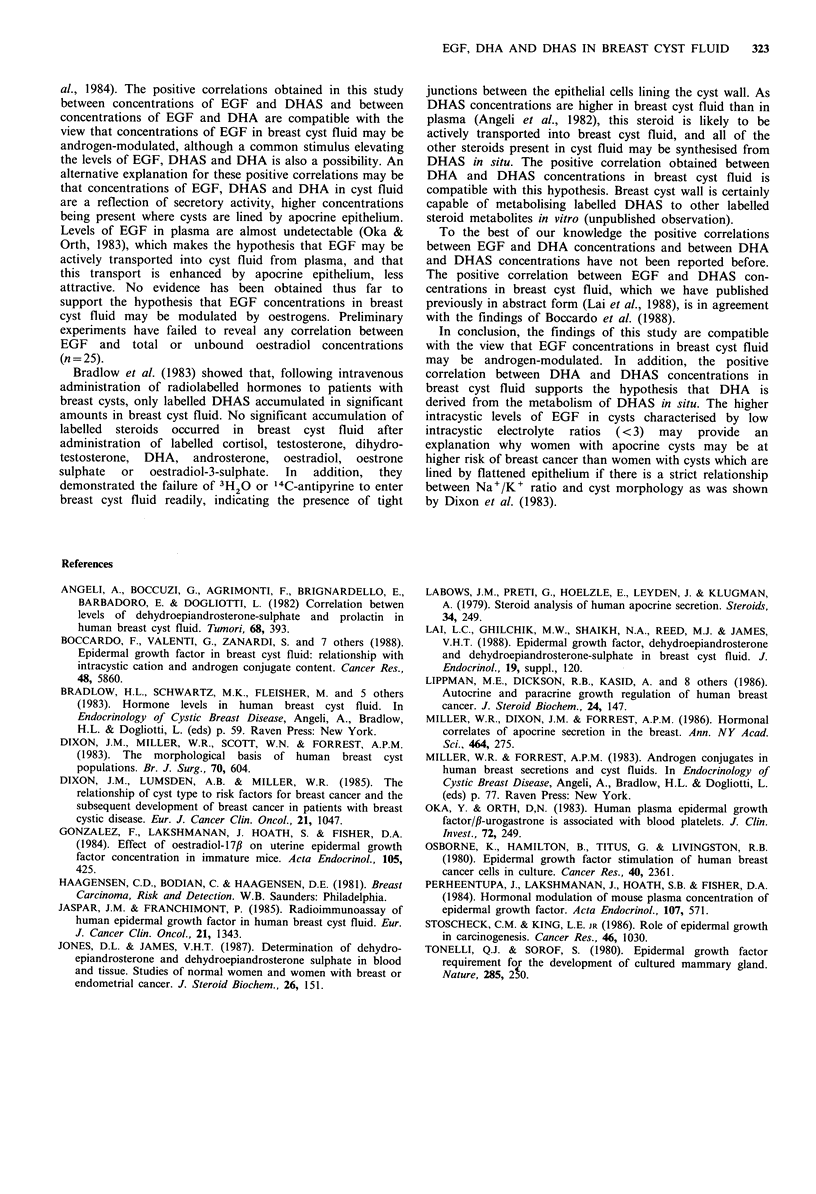# Relationship between epidermal growth factor and dehydroepiandrosterone and its sulphate in breast cyst fluid.

**DOI:** 10.1038/bjc.1989.278

**Published:** 1989-09

**Authors:** L. C. Lai, M. W. Ghilchik, N. A. Shaikh, M. J. Reed, V. H. James

**Affiliations:** Department of Chemical Pathology, St Mary's Hospital Medical School, London, UK.

## Abstract

Gross cystic breast disease is a common condition. Women with apocrine breast cysts may be at higher risk of breast cancer than women with cysts which are lined by flattened epithelium. Apocrine cysts have been shown to be associated with higher intracystic levels of dehydroepiandrosterone sulphate and intracystic sodium to potassium ratios of less than 3. In this study we measured the concentrations of epidermal growth factor, dehydroepiandrosterone and its sulphate in breast cyst fluid. The concentrations of all three analytes were significantly higher in cysts with intracystic Na+/K+ ratios of less than 3 than in cysts with electrolyte ratios of greater than or equal to 3 (P less than 0.001). The higher levels of EGF in cysts with low intracystic electrolyte ratios may provide an explanation of why women with apocrine cysts may be at greater risk of breast cancer. Positive correlations were obtained between concentrations of EGF and DHAS and between EGF and DHA, compatible with the view that intracystic EGF levels may be androgen-modulated. A positive correlation was also obtained between DHA and DHAS concentrations which supports the view that DHA in cyst fluid may be derived from the metabolism of DHAS in the breast cyst wall.


 © The Macmillan Press Ltd., 1989

### Relationship between epidermal growth factor and dehydroepiandrosterone and its sulphate in breast cyst fluid

L.C. Lai[1], M.W. Ghilchik[2], N.A. Shaikh[2], M.J. Reed[1] & V.H.T. James[1]

[1]*Department of Chemical Pathology, St Mary's Hospital Medical School, London W2 1PG, and* [2]*Breast Clinic, St Mary's Hospital, London W2 1NY, UK.*

**Summary** Gross cystic breast disease is a common condition. Women with apocrine breast cysts may be at higher risk of breast cancer than women with cysts which are lined by flattened epithelium. Apocrine cysts have been shown to be associated with higher intracystic levels of dehydroepiandrosterone sulphate and intracystic sodium to potassium ratios of less than 3. In this study we measured the concentrations of epidermal growth factor, dehydroepiandrosterone and its sulphate in breast cyst fluid. The concentrations of all three analytes were significantly higher in cysts with intracystic $Na^+/K^+$ ratios of less than 3 than in cysts with electrolyte ratios of greater than or equal to 3 ($P<0.001$). The higher levels of EGF in cysts with low intracystic electrolyte ratios may provide an explanation of why women with apocrine cysts may be at greater risk of breast cancer. Positive correlations were obtained between concentrations of EGF and DHAS and between EGF and DHA, compatible with the view that intracystic EGF levels may be androgen-modulated. A positive correlation was also obtained between DHA and DHAS concentrations which supports the view that DHA in cyst fluid may be derived from the metabolism of DHAS in the breast cyst wall.

Gross cystic breast disease (cysts more than 3 mm in diameter) is a common condition affecting about 7% of women in the western world (Haagensen *et al.*, 1981). Dixon *et al.* (1983) have shown that these cysts can be divided into two groups according to breast cyst fluid sodium to potassium ratios, namely, $Na^+/K^+<3$ (cysts lined by apocrine epithelium) and $Na^+/K^+\geqslant 3$ (cysts lined by flattened epithelium). Women with apocrine breast cysts may be at a higher risk of subsequent development of breast cancer than women with cysts which are lined by flattened epithelium (Haagensen *et al.*, 1981; Dixon *et al.*, 1985).

Growth factors such as epidermal growth factor (EGF) may be implicated in the development of human cancer (Stoscheck & King, 1986). Wide-ranging concentrations of EGF have been found in breast cyst fluid (Jaspar & Franchimont, 1985). EGF has been shown to stimulate the proliferation of mammary epithelial cells (Tonelli & Sorof, 1980) and human breast cancer cells in culture (Osborne *et al.*, 1980).

Dehydroepiandrosterone (DHA) and its sulphate (DHAS) are also present in wide-ranging concentrations in breast cyst fluid (Bradlow *et al.*, 1983; Miller & Forrest, 1983). DHAS is a marker of apocrine activity being present in high concentrations in apocrine secretions (Labows *et al.*, 1979). Miller *et al.* (1986) found breast cyst fluid DHAS levels to be significantly higher in apocrine cysts than in cysts lined by flattened epithelium.

Since EGF production in some tissues is sensitive to sex hormones, EGF levels in breast cyst fluid may be androgen-modulated. This study was, therefore, designed to assess the relationship between intracystic concentrations of EGF and DHAS and between EGF and DHA.

### Materials and methods

### *Patient samples*

Needle aspiration of breast cysts was carried out as a diagnostic procedure. The cyst fluid was centrifuged at 1,500 *g* for 10 min and the supernatant was stored at −20°C until assayed. EGF was assayed on 105 cyst fluid samples. DHAS and DHA were assayed on a smaller number of samples (49 and 71 samples respectively), the remaining samples being used in various other experiments. Cytological examination of cyst fluid was not performed. Approval for this study was obtained from the local district ethics committee.

### *Materials*

Anti-human EGF antiserum, raised in Dutch male rabbits, and pure urogastrone (EGF isolated from urine) were gifts from Dr H. Gregory (ICI plc, Macclesfield, UK). Recombinant human EGF, iodine-125 and 1,2,6,7-$^3$H-DHA (87 Ci mmol$^{-1}$) were obtained from Amersham International plc (Amersham, Bucks, UK); 1,2,6,7,-$^3$H-DHAS, ammonium salt (92 Ci mmol$^{-1}$), was purchased from DuPont (UK) Ltd (NEN Products Division, Wedgewood Way, Stevenage, Herts, UK). Anti-DHA antiserum was raised in rabbits against DHA-7-carboxymethyloxime-bovine serum albumin.

### *Measurement of electrolytes*

Sodium and potassium concentrations in cyst fluid were measured by an indirect ion-selective electrode (Beckman Electrolyte 2 Analyser).

### *Measurement of EGF*

Tracer was prepared by iodination of recombinant human EGF using chloramine T and purified on a Sephadex G-25 medium column.

The buffer used for the radioimmunoassay was 0.05 M phosphate buffer, pH 7.4, containing 0.2% w/v bovine serum albumin (RIA grade) and 0.1% w/v sodium azide. Assays were performed in duplicate. Non-specific binding tubes and total counts were included in each assay. Standards were prepared from pure urogastrone in assay buffer.

An aliquot (0.1 ml) of standard (0, 0.05, 0.1, 0.2, 0.39, 0.78, 1.56, 3.12 and 6.25 ng ml$^{-1}$) or sample was pre-incubated with 0.1 ml of antiserum (working dilution of 1 in 30,000 with a zero binding of 30%) diluted in assay buffer containing non-immune rabbit serum (4 µl ml$^{-1}$) and 0.2 ml of assay buffer overnight at 4°C. An aliquot (0.1 ml) of labelled antigen (about 8,000 c.p.m.) was then added and the tubes were incubated overnight at 4°C for 2 days. Donkey anti-rabbit immunoglobulin G (0.1 ml) was added and the tubes incubated overnight at 4°C. The tubes were centrifuged at 1,500 *g* for 15 min at 4°C and the pellets counted for 2 min each on a gamma counter.

The sensitivity of this assay was determined from the precision of 10 pairs of zero standards (2 s.d. of the mean

Correspondence: L.C. Lai.
Received 20 January 1989, and in revised form, 22 March 1989.

zero binding) and was 0.04 ng ml$^{-1}$. The intra-assay coefficient of variation (c.v.), calculated using the difference between duplicates, was 8.6% ($n=20$ pairs). The inter-assay c.v. was 7.4% at a concentration of 84.6 ng ml$^{-1}$ ($n=5$).

Breast cyst fluid was assayed in several dilutions and the competitive binding curves for the sample dilutions were parallel to that of the standard curve.

*Measurement of DHA and DHAS*

The methodology for the measurement of DHA and DHAS has been described by Jones and James (1987).

The sensitivity of the DHA assay was 2 nmol l$^{-1}$, the intra-assay c.v., determined from 10 duplicates, was 12% and the inter-assay c.v. for a concentration of 12.8 nmol l$^{-1}$ was 12.6% ($n=5$).

The sensitivity of the DHAS assay was 0.24 $\mu$mol l$^{-1}$, the intra-assay c.v., determined from 12 dupicates, was 4.1% and the inter-assay c.v. for a concentration of 4.85 $\mu$mol l$^{-1}$ was 8.7% ($n=6$).

The mean recoveries ($\pm 1$ s.d.) after thin layer chromatography were 79.4 $\pm$ 7.9% ($n=20$) for the DHA assay and 65.6 $\pm$ 7.3% ($n=27$) for the DHAS assay.

*Statistical analyses*

The distributions of the various analytes were not Gaussian. Non-parametric statistics were, therefore, used. Wilcoxon's rank sum test was used to compare differences between the distributions of the various analytes in the two groups of breast cysts as defined by their sodium to potassium ratios. Correlations were assessed using Spearman's rank correlation method (Spearman's rank correlation coefficient $= r_s$). Results were regarded to be statistically significant when $P<0.05$.

### Results

The frequency polygon of log $_{10}$ $Na^+/K^+$ shows a bimodal distribution (Figure 1). A cut-off point of $Na^+/K^+=3$, which appeared to reasonably separate the two groups of breast cysts, was arbitrarily chosen.

EGF concentrations in the low electrolyte ratio group, i.e. $Na^+/K^+<3$ (median = 319 ng ml$^{-1}$, $n=66$), were significantly higher than the concentrations in the high electrolyte ratio group, i.e. $Na^+/K^+ \geqslant 3$ (median = 71 ng ml$^{-1}$, $n=39$), $P<0.0003$ (Figure 2).

Figure 1 Frequency polygon of $\log_{10}$ $Na^+/K^+$. The dotted line represents the cut-off between the two groups of breast cysts.

Figure 3 shows the concentrations of DHAS in the two cyst groups. DHAS levels in the low electrolyte ratio group (median = 155 $\mu$mol l$^{-1}$, $n=31$) were significantly higher than

Figure 2 EGF concentrations in the two groups of breast cysts. The horizontal lines represent median concentrations in each cyst group: 319 ng ml$^{-1}$ ($n=66$) in the low electrolyte ratio group and 71 ng ml$^{-1}$ ($n=39$) in the high electrolyte ratio group. $P<0.0003$ between groups.

Figure 3 DHAS concentrations in the two groups of breast cysts. The horizontal lines represent median concentrations in each cyst group: 155 $\mu$mol l$^{-1}$ ($n=31$) in the low electrolyte ratio group and 16 $\mu$mol l$^{-1}$ ($n=18$) in the high electrolyte ratio group. $P<0.001$ between groups.

in the high electrolyte ratio group (median = 16 $\mu mol\,l^{-1}$, $n = 18$), $P < 0.001$.

The concentrations of DHA in the low electrolyte ratio group (median = 22 $nmol\,l^{-1}$, $n = 45$) were also significantly higher than in the high electrolyte ratio group (median = 10.7 $nmol\,l^{-1}$, $n = 26$), $P < 0.001$ (Figure 4).

Figure 5 shows EGF concentrations versus DHAS con-

**Figure 4** DHA concentrations in the two groups of breast cysts. The horizontal lines represent median concentrations in each cyst group: 22 $nmol\,l^{-1}$ ($n = 45$) in the low electrolyte ratio group and 10.7 $nmol\,l^{-1}$ ($n = 26$) in the high electrolyte ratio group. $P < 0.001$ between groups.

**Figure 5** EGF concentration versus DHAS concentration ($n = 49$, $r_s = 0.81$, $P < 0.001$).

**Figure 6** EGF concentration versus DHA concentration ($n = 71$, $r_s = 0.48$, $P < 0.001$).

**Figure 7** DHA concentration versus DHAS concentration ($n = 48$, $r_s = 0.76$, $P < 0.001$).

centrations. A positive correlation was obtained between the two analytes ($n = 49$, $r_s = 0.81$, $P < 0.001$).

Figure 6 shows that a weaker positive correlation was obtained between EGF and DHA concentrations ($n = 71$, $r_s = 0.48$, $P < 0.001$).

A positive correlation ($n = 48$, $r_s = 0.76$, $P < 0.001$) was obtained between concentrations of DHA and DHAS in breast cyst fluid (Figure 7).

### Discussion

Testosterone has been shown to increase serum and submaxillary salivary gland levels of EGF (Perheentupa *et al.*, 1984) and oestradiol to increase EGF-related polypeptide production by various human cancer cell lines (Lippman *et al.*, 1985). In addition, oestradiol has been shown to increase uterine EGF concentrations in immature mice (Gonzalez *et*

*al.*, 1984). The positive correlations obtained in this study between concentrations of EGF and DHAS and between concentrations of EGF and DHA are compatible with the view that concentrations of EGF in breast cyst fluid may be androgen-modulated, although a common stimulus elevating the levels of EGF, DHAS and DHA is also a possibility. An alternative explanation for these positive correlations may be that concentrations of EGF, DHAS and DHA in cyst fluid are a reflection of secretory activity, higher concentrations being present where cysts are lined by apocrine epithelium. Levels of EGF in plasma are almost undetectable (Oka & Orth, 1983), which makes the hypothesis that EGF may be actively transported into cyst fluid from plasma, and that this transport is enhanced by apocrine epithelium, less attractive. No evidence has been obtained thus far to support the hypothesis that EGF concentrations in breast cyst fluid may be modulated by oestrogens. Preliminary experiments have failed to reveal any correlation between EGF and total or unbound oestradiol concentrations ($n=25$).

Bradlow *et al.* (1983) showed that, following intravenous administration of radiolabelled hormones to patients with breast cysts, only labelled DHAS accumulated in significant amounts in breast cyst fluid. No significant accumulation of labelled steroids occurred in breast cyst fluid after administration of labelled cortisol, testosterone, dihydrotestosterone, DHA, androsterone, oestradiol, oestrone sulphate or oestradiol-3-sulphate. In addition, they demonstrated the failure of $^{3}H_{2}O$ or $^{14}C$-antipyrine to enter breast cyst fluid readily, indicating the presence of tight junctions between the epithelial cells lining the cyst wall. As DHAS concentrations are higher in breast cyst fluid than in plasma (Angeli *et al.*, 1982), this steroid is likely to be actively transported into breast cyst fluid, and all of the other steroids present in cyst fluid may be synthesised from DHAS *in situ*. The positive correlation obtained between DHA and DHAS concentrations in breast cyst fluid is compatible with this hypothesis. Breast cyst wall is certainly capable of metabolising labelled DHAS to other labelled steroid metabolites *in vitro* (unpublished observation).

To the best of our knowledge the positive correlations between EGF and DHA concentrations and between DHA and DHAS concentrations have not been reported before. The positive correlation between EGF and DHAS concentrations in breast cyst fluid, which we have published previously in abstract form (Lai *et al.*, 1988), is in agreement with the findings of Boccardo *et al.* (1988).

In conclusion, the findings of this study are compatible with the view that EGF concentrations in breast cyst fluid may be androgen-modulated. In addition, the positive correlation between DHA and DHAS concentrations in breast cyst fluid supports the hypothesis that DHA is derived from the metabolism of DHAS *in situ*. The higher intracystic levels of EGF in cysts characterised by low intracystic electrolyte ratios ($<3$) may provide an explanation why women with apocrine cysts may be at higher risk of breast cancer than women with cysts which are lined by flattened epithelium if there is a strict relationship between $Na^{+}/K^{+}$ ratio and cyst morphology as was shown by Dixon *et al.* (1983).

### References

ANGELI, A., BOCCUZI, G., AGRIMONTI, F., BRIGNARDELLO, E., BARBADORO, E. & DOGLIOTTI, L. (1982) Correlation betwen levels of dehydroepiandrosterone-sulphate and prolactin in human breast cyst fluid. *Tumori*, **68**, 393.

BOCCARDO, F., VALENTI, G., ZANARDI, S. and 7 others (1988). Epidermal growth factor in breast cyst fluid: relationship with intracystic cation and androgen conjugate content. *Cancer Res.*, **48**, 5860.

BRADLOW, H.L., SCHWARTZ, M.K., FLEISHER, M. and 5 others (1983). Hormone levels in human breast cyst fluid. In *Endocrinology of Cystic Breast Disease*, Angeli, A., Bradlow, H.L. & Dogliotti, L. (eds) p. 59. Raven Press: New York.

DIXON, J.M., MILLER, W.R., SCOTT, W.N. & FORREST, A.P.M. (1983). The morphological basis of human breast cyst populations. *Br. J. Surg.*, **70**, 604.

DIXON, J.M., LUMSDEN, A.B. & MILLER, W.R. (1985). The relationship of cyst type to risk factors for breast cancer and the subsequent development of breast cancer in patients with breast cystic disease. *Eur. J. Cancer Clin. Oncol.*, **21**, 1047.

GONZALEZ, F., LAKSHMANAN, J. HOATH, S. & FISHER, D.A. (1984). Effect of oestradiol-17$\beta$ on uterine epidermal growth factor concentration in immature mice. *Acta Endocrinol.*, **105**, 425.

HAAGENSEN, C.D., BODIAN, C. & HAAGENSEN, D.E. (1981). *Breast Carcinoma, Risk and Detection.* W.B. Saunders: Philadelphia.

JASPAR, J.M. & FRANCHIMONT, P. (1985). Radioimmunoassay of human epidermal growth factor in human breast cyst fluid. *Eur. J. Cancer Clin. Oncol.*, **21**, 1343.

JONES, D.L. & JAMES, V.H.T. (1987). Determination of dehydroepiandrosterone and dehydroepiandrosterone sulphate in blood and tissue. Studies of normal women and women with breast or endometrial cancer. *J. Steroid Biochem.*, **26**, 151.

LABOWS, J.M., PRETI, G., HOELZLE, E., LEYDEN, J. & KLUGMAN, A. (1979). Steroid analysis of human apocrine secretion. *Steroids*, **34**, 249.

LAI, L.C., GHILCHIK, M.W., SHAIKH, N.A., REED, M.J. & JAMES, V.H.T. (1988). Epidermal growth factor, dehydroepiandrosterone and dehydroepiandrosterone-sulphate in breast cyst fluid. *J. Endocrinol.*, **19**, suppl., 120.

LIPPMAN, M.E., DICKSON, R.B., KASID, A. and 8 others (1986). Autocrine and paracrine growth regulation of human breast cancer. *J. Steroid Biochem.*, **24**, 147.

MILLER, W.R., DIXON, J.M. & FORREST, A.P.M. (1986). Hormonal correlates of apocrine secretion in the breast. *Ann. NY Acad. Sci.*, **464**, 275.

MILLER, W.R. & FORREST, A.P.M. (1983). Androgen conjugates in human breast secretions and cyst fluids. In *Endocrinology of Cystic Breast Disease*, Angeli, A., Bradlow, H.L. & Dogliotti, L. (eds) p. 77. Raven Press: New York.

OKA, Y. & ORTH, D,N. (1983). Human plasma epidermal growth factor/$\beta$-urogastrone is associated with blood platelets. *J. Clin. Invest.*, **72**, 249.

OSBORNE, K., HAMILTON, B., TITUS, G. & LIVINGSTON, R.B. (1980). Epidermal growth factor stimulation of human breast cancer cells in culture. *Cancer Res.*, **40**, 2361.

PERHEENTUPA, J., LAKSHMANAN, J., HOATH, S.B. & FISHER, D.A. (1984). Hormonal modulation of mouse plasma concentration of epidermal growth factor. *Acta Endocrinol.*, **107**, 571.

STOSCHECK, C.M. & KING, L.E. JR (1986). Role of epidermal growth in carcinogenesis. *Cancer Res.*, **46**, 1030.

TONELLI, Q.J. & SOROF, S. (1980). Epidermal growth factor requirement for the development of cultured mammary gland. *Nature*, **285**, 250.